# Exogenous IL-25 ameliorates airway neutrophilia via suppressing macrophage M1 polarization and the expression of IL-12 and IL-23 in asthma

**DOI:** 10.1186/s12931-023-02557-5

**Published:** 2023-10-28

**Authors:** Chenli Chang, Gongqi Chen, Wenliang Wu, Dian Chen, Shengchong Chen, Jiali Gao, Yuchen Feng, Guohua Zhen

**Affiliations:** 1 Division of Pulmonary and Critical Care Medicine, Department of Internal Medicine, Tongji Hospital, Tongji Medical College, Huazhong University of Science and Technology, Wuhan, China; 2 Key Laboratory of Respiratory Diseases, National Clinical Research Center for Respiratory Diseases, National Health Commission of People’s Republic of China, Wuhan, China; 3 Division of Pulmonary and Critical Care Medicine, Tongji Hospital, 1095 Jiefang Avenue, 430030 Wuhan, China; 4 Division of Respiratory and Critical Care Medicine, Tongji Hospital, 430030 Wuhan, China

**Keywords:** Asthma, Airway inflammation, Neutrophilia, Macrophage polarization, IL-12, IL-23, IL-25

## Abstract

**Background:**

Severe asthma is associated with substantial mortality and has unmet therapeutic need. A subset of severe asthma is characterized by neutrophilic airway inflammation. Classically activated (or M1) macrophages which express IL-12 and IL-23 are associated with airway neutrophilia in asthma. Exogenous IL-25 was reported to suppress intestinal inflammation in animal models of inflammatory bowel diseases via suppressing IL-12 and IL-23 production. We hypothesize that IL-25 ameliorates airway neutrophilia via inhibiting macrophage M1 polarization and the expression of IL-12 and IL-23 in asthma.

**Methods:**

In a mouse model of neutrophil-dominant allergic airway inflammation, the effect of mouse recombinant IL-25 on airway inflammation were assessed by H&E staining and bronchoalveolar lavage (BAL) cell counting. The percentage of M1 macrophages in lung tissue and BAL cells were analyzed by flow cytometry. Quantitative PCR and immunostaining were performed to measure the expression of *Il12, Il23*, and inflammatory cytokines. Mechanistic experiments were performed in primary culture of macrophages from mouse lungs. The expression of IL-12, IL-23 and IL-25 in sputum was analyzed in a cohort of severe asthma and subjects with eosinophilic or non-eosinophilic asthma.

**Results:**

Intranasal administration of IL-25 markedly decreased the number of neutrophils in BAL cells in a murine model of neutrophil-dominant allergic airway inflammation. Moreover, exogenous IL-25 decreased the number of M1 macrophages, and reduced the expression of IL-12, IL-23 in the lungs of the mouse model. Exogenous IL-25 also inhibited the expression of inflammatory cytokines IL-1β, IFN-γ, TNF-α and IL-17 A. In vitro, IL-25 suppressed IL-12 and IL-23 expression in lipopolysaccharide (LPS)-stimulated primary culture of mouse pulmonary macrophages. Mechanistically, IL-25 inhibited LPS-induced c-Rel translocation to nucleus via STAT3-dependent signaling. In a cohort of severe asthma, IL-25 protein levels in sputum were significantly lower than control subjects. The transcript levels of *IL-12* and *IL-23* were increased whereas IL-25 transcripts were decreased in sputum cells from subjects with non-eosinophilic asthma compared to eosinophilic asthma.

**Conclusions:**

IL-25 expression is downregulated in subjects with severe or non-eosinophilic asthma. Exogenous IL-25 ameliorates airway neutrophilia, at least in part, via inhibiting macrophage M1 polarization and the expression of IL-12 and IL-23.

**Supplementary Information:**

The online version contains supplementary material available at 10.1186/s12931-023-02557-5.

## Background

Asthma affects ~ 300 million people worldwide, and approximately 25,000 people died each year from asthma [1]. Severe asthma is a major challenge because of substantial mortality and the poor responsiveness to treatment including corticosteroids. Patients with severe asthma require frequent hospitalization and emergency care, which accounts for nearly 50% of the healthcare cost associated with asthma [2]. Severe asthma is a heterogeneous condition [3]. Several studies identified a similar subgroup with late-onset nonallergic neutrophilic severe asthma [4–6]. Neutrophilic or non-eosinophilic airway inflammation is often associated with severe asthma that does not respond to steroids [7, 8]. Thus, airway neutrophilia is an essential biomarker for a subset of severe asthma.

Macrophages are abundant immunocytes in the lung and play critical roles in the pathogenesis of asthma [9]. Classically activated (or M1) macrophages and alternatively activated (or M2) macrophages are involved in pro-inflammatory and anti-inflammatory processes, respectively [10]. It was reported that the percentage of M1 macrophages in sputum was higher in non-eosinophilic asthma compared to eosinophilic asthma. Moreover, the percentage of M1 macrophages was positively correlated with that of neutrophils in sputum from asthma patients [11]. This suggests that macrophage M1 polarization is associated with airway neutrophilia in asthma. M1 macrophages can produce proinflammatory cytokines IL-12 and IL-23 which promote the differentiation of Th1 and Th17 cells and neutrophilic inflammation [12–14]. *IL-12B* mRNA expression was increased in BAL cells from patients with severe asthma [15]. Recently, it has been reported that IL-12 and IL-23 expression was increased in the lung of a mouse model of corticosteroid-resistant severe asthma [16]. IL-12 is a heterodimer of the subunits p35 (encoded by *IL-12A*) and p40 (encoded by *IL-12B*), while IL-23 is composed of p19 (encoded by *IL-23A*) and p40 [17]. The expression of IL-12 p40, a shared subunit of both cytokines, is selectively controlled by c-Rel in dendritic cells and macrophages [18, 19].

It has been reported that the expression of *Il12b* and *Il23a* was increased in the large intestine of *Il25*^−/−^ mice compared to WT mice [20]. *Il25*-deficient mice developed intestinal inflammation characterized by exaggerated interferon (IFN)-γ and IL-17 production after parasite infection [21]. Interestingly, exogenous IL-25 suppresses Th17 cell expansion by inhibiting the expression of macrophage-derived IL-23 [20]. IL-25 suppresses the expression of IL-12 and IL-23 in CD14^+^ cells from patients with Crohn’s disease. Moreover, IL-25 ameliorates the inflammation in multiple mouse models of colitis [22]. These reports suggest that IL-25 can suppress IL-12 and IL-23-mediated inflammation. Given that M1 macrophages are associated with airway neutrophilia and express IL-12 and IL-23 [11, 13], we hypothesize that IL-25 ameliorates airway neutrophilia via suppressing macrophage M1 polarization and the expression of IL-12 and IL-23 in asthma.

To test the hypothesis, we used exogenous IL-25 to treat a mouse model of neutrophil-dominant allergic airway inflammation. We found that exogenous IL-25 indeed decreased airway neutrophilia and airway hyperresponsiveness in the mouse model. Moreover, macrophage M1 polarization and the expression of IL-12, IL-23 was suppressed by IL-25 treatment. Of note, IL-25 expression in sputum were decreased in subjects with severe asthma or non-eosinophilic asthma. However, the expression of IL-12 and IL-23 and M1 macrophage markers were increased in sputum cells from patients with non-eosinophilic asthma.

## Materials and methods

### Subjects

We recruited 12 control subjects, 23 eosinophilic asthma, and 17 non-eosinophilic asthma patients. All subjects were Chinese and recruited from Tongji Hospital. Asthma patients had symptoms of recurrent episodes of wheezing, breathlessness, chest tightness, and coughing, and had accumulated dosage of methacholine provoking a 20% fall (PD_20_) of forced expiratory volume in the first second (FEV_1_) < 2.505 mg and/or ≥ 12% increase in FEV_1_ following inhalation of 200 µg salbutamol. Healthy control subjects had no respiratory symptoms, normal spirometric value, and methacholine PD_20_ ≥ 2.505 mg. None of the subjects had ever smoked or received inhaled or oral corticosteroid or leukotriene antagonists. For each subject, we recorded demographic information, collected induced sputum, and measured spirometry. We performed bronchoscopy with endobronchial brushing and biopsy. Biopsy techniques and methods for spirometry and FeNO measurement were described previously [23]. Written informed consent was obtained from all subjects. The ethics committee of Tongji Hospital, Huazhong University of Science and Technology, approved the study.

### Murine models of Asthma

Six to eight-week-old female C57BL/6J mice (Beijing Vital River Laboratory Animal Technology Co., Ltd.) were divided into four groups (6 to 8 mice in each group): (1) control group; (2) OVA group; (3) OVA/ LPS group; (4) OVA/ LPS/ IL-25 group. Mice in the OVA group were sensitized by intraperitoneal injection of 200 µL normal saline solution containing 100 µg OVA and 100 µL aluminum hydroxide on day 0, 7 and 14 respectively, and challenged by intranasal drip of 40 µL normal saline containing 1 mg OVA on day 21, 22 and 23; mice in OVA / LPS group were sensitized with intranasal administration of 75 µg OVA and 10 µg LPS in 40 µL saline on day 0, 1, 2 and 7, and challenged with intranasal administration of 50 µg OVA in 40 µL saline on day 14, 15, 21 and 22; mice in the control group were sensitized with an equal volume of normal saline; mice in OVA/ LPS/ IL-25 group were sensitized with 0.4 µg IL-25 recombinant protein was intranasally instilled at -1, 6, 13 and 20 days, and the other modeling methods were the same as those in OVA / LPS group. Twenty-four hours after the last challenge of OVA, we measured pulmonary resistance in response to a range of concentrations of intravenous methacholine using the forced oscillation technique with the FlexiVent system (SCIREQ). Lung tissues were collected for histological analysis, quantitative PCR, and immunostaining. Animal experiments were approved by the ethics committee of Tongji Hospital, Huazhong University of Science and Technology.

### Cell culture and treatment

Mouse pulmonary macrophages were isolated from lung tissue of C57BL/6J mice aged around 6-week-old, the mice were euthanized and their lung tissues were removed for enzymatic digestion and processed into single cell suspension. After centrifugation, the cell sediment was removed, and the cells were resuspended in RPMI-1640 medium (Hyclone) with 10% FBS (Gibco), 100 IU/mL penicillin, and 100 mg/mL streptomycin and cell cultures were maintained at 37 °C and 5% CO_2_ for 1 h. The medium was discarded and washed with PBS to remove the suspended cells. The culture was replaced with complete medium overnight, and Giemsa staining confirmed that more than 95% of the adherent cells were lung macrophages. Based on the above steps, approximately 1*10^6 macrophages can be extracted from each mouse lung tissue, which can be used for inoculation with a six well plate. Escherichia coli–derived LPS (1 µg/mL; Sigma-Aldrich), IL-25 (50 ng/mL, Biolegend), Stattic (5 µM, MedChemExpress) were used when indicated.

### Quantitative PCR

Total RNA from bronchial epithelial brushing, mouse lungs, and cultured cells was isolated using TRIzol (Takara) and reverse-transcribed using the PrimeScript RT reagent kit (Takara). The expression of each gene was determined using a BioRad CFX96™ System (Biorad). The primers used are listed in Table S1. The gene expression was determined by the 2 − ΔΔCT method. The gene expression was expressed as log2 transformed and relative to the median of healthy control subjects or the mean of the control group.

### Histology

Human bronchial biopsy and mouse lung sections were stained with hematoxylin and eosin (H&E). Observers who were blinded to the clinical status of the subjects counted numbers of eosinophils/mm2 submucosa as previously described. The methods for assessment of airway inflammation were described previously [24].

### Western blotting

In protein sample preparation, the supernatant of cell culture was collected and the cells were lysed in ice-cold RIPA lysis buffer (Beyotime) with EDTA-free Protease Inhibitor Cocktail Tablets (Roche). The protein samples were separated on a 10% SDS-PAGE or non-reducing SDS-PAGE and electrophoretically transferred to a PVDF membrane (Millipore, Billerica, MA). After they were blocked in Tris-buffered saline (pH 7.4) containing Tween-20 (0.1%) (Sigma-Aldrich) with 5% (w/v) nonfat milk, the membranes were incubated in 5% milk with the antibodies specific for the proteins (rabbit anti-IL-12 A mAb, 1:2000, anti-IL-12B mAb, 1:2000,anti-cRel mAb,1:2000, Boster, Wuhan, China; rabbit anti-STAT3 mAb, 1:1000, anti-pSTAT3 mAb, 1:1000,CST,USA; mouse anti-β-Tubulin mAb, 1:4000, Sungene Biotech, Tianjin, China; mouse anti-GAPDH mAb, 1:2000, anti-PCNA mAb, 1:2000, Abbkine Scientific,USA), separately, overnight at 4 °C. After that, the membranes were exposed to horseradish peroxidase-conjugated goat anti-rabbit/mouse secondary antibody (1:5000, Servicebio, Wuhan, China) for 1 h at room temperature. Finally, the signals were detected using an ECL kit (MCE) according to the manufacturer’s instructions.

### Flow cytometry

Following euthanasia the lung samples of mice were cut into small pieces with scissors in Hank’s balanced salt solution (HBSS) buffer. Single-cell suspensions were prepared by filtering through a 100 μm nylon filter strainer and washed thoroughly in HBSS) buffer supplemented with 2% FBS, 20 mM HEPES, and 5 mM EDTA. The cells were resuspended according to 1*10^6 cells/100 µL to prepare single-cell suspension. 100 µL cell suspension was added into the flow tube, and 2 µL FcBlock was added into each tube, and incubated on ice for 10 min. Blank tube, single staining tube, and sample tube were set respectively. A single antibody was added into the single staining tube. 0.5 µL LIVE/DEAD™ Fixable Yellow Dead Cell Stain Kit (Invitrogen, USA), 2 µL anti-CD45 (bv605), 2.5 µL anti-F4/80 (PE) and 5 µL anti-CD86 (percp/cy5.5) antibodies were added into the sample tube and incubate on ice for 15-20 min, and keep away from light. After permeabilization with intracellular Staining Permeabilization Wash Buffer (Biolegend), 1.5 µL of anti-cd206 (APC) antibody was added and incubated for 20 min. Cells were suspended by PBS and detected as soon as possible.

### Immunofluorescence

The frozen slides were taken out from the refrigerator at − 80 ℃ and placed at room temperature, rewarming for 30 min, PBS washing for 3 times, 5 min each time. A circle was drawn around the tissue with a histochemical pen to make the antibody cover the tissue effectively. Donkey Serum containing 1% BSA and 1‰ Triton-x 100 was dripped to cover the tissue and incubated at room temperature for 30–60 min. After enough sealing time, the blocking solution was gently shaken off, and the tissue was directly incubated with IL-12B (1:200) and CD80 (1:100) antibodies in a wet box overnight at 4 ℃. After being washed with PBS, the slices were incubated with fluorescently labeled anti-rabbit secondary antibody at room temperature for 1 h. DAPI staining of nuclei after PBS washing. Washing again, the slices were sealed with an anti-fluorescence quenching sealing agent. Then, slices were observed under a fluorescence microscope within one week to avoid fluorescence quenching, and images were collected.

### Statistical analysis

We analyzed data using Prism version 8 (GraphPad Software). For normally distributed data, we calculated means ± standard deviation (SD) and used parametric tests (Student’s t-test or one-way ANOVA followed by Tukey’s multiple comparison test) to compare across groups. For non‐normally distributed data, we calculated medians with interquartile ranges and used non‐parametric tests (Mann–Whitney test). We analyzed correlation using Spearman’s rank‐order correlation. P < 0.05 was considered statistically significant.

## Results

### Exogenous IL-25 inhibits airway neutrophilia and airway hyperresponsiveness in a mouse model of neutrophilia-dominant airway inflammation

**Fig. 1:**
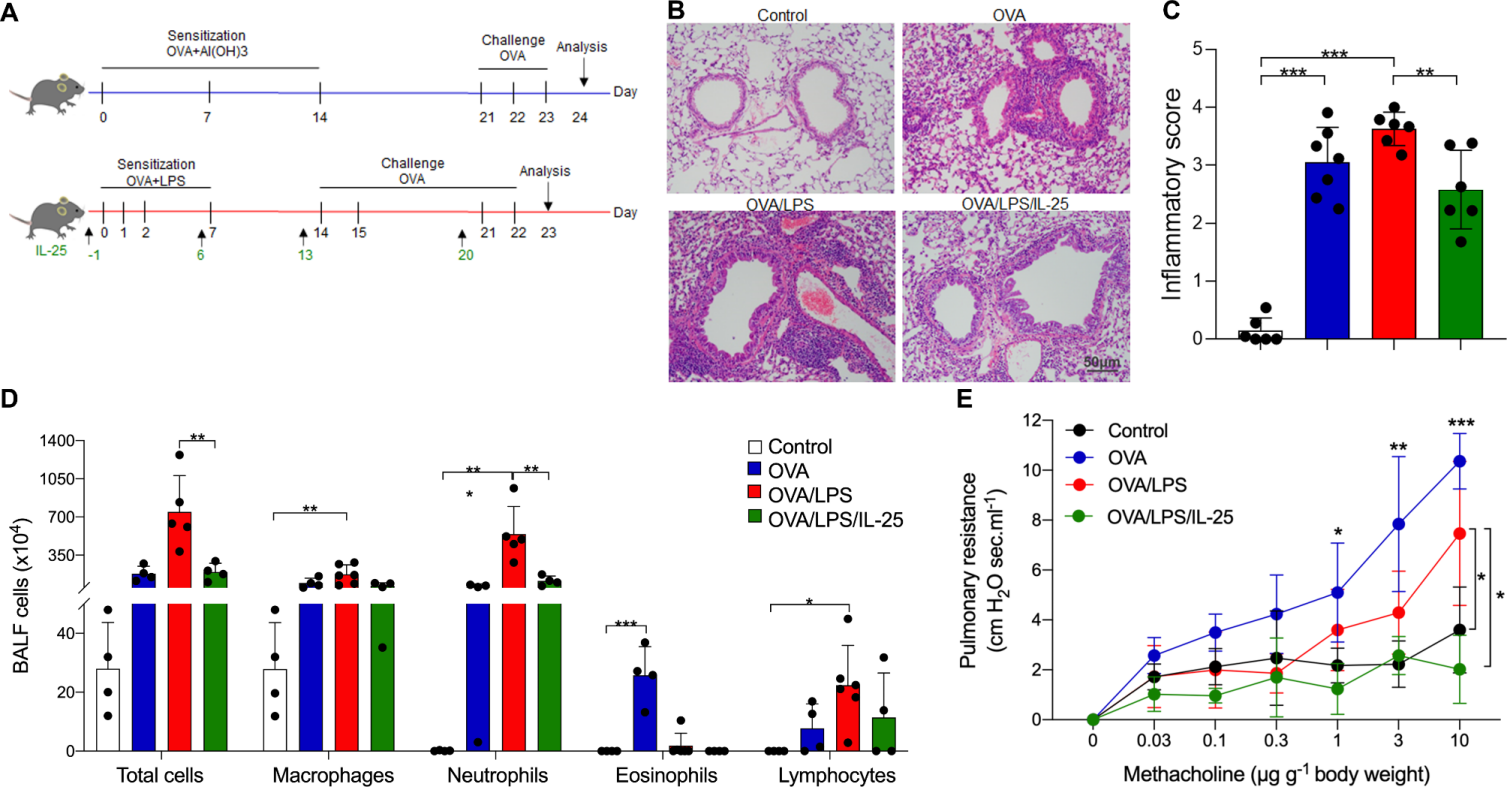
Exogenous IL-25 inhibited airway neutrophilic inflammation in a mouse model of neutrophilia-dominant airway inflammation. (**A**) Protocol of the mouse models and IL-25 intervention. (**B**) Representative images of H&E staining of mouse lung sections. Scale bar, 100 μm. (**C**) Assessment of the airway inflammation of the mice using lung inflammatory scores described in Methods. (**D**) The number of inflammatory cells in BALF of mice. E, Pulmonary resistance in response to different concentrations of intravenous methacholine. There were 4–6 mice in each group. One-way ANOVA was used for statistical analysis (* P < 0.05; ** P < 0.01; *** P < 0.001)

Two mouse models of allergic airway disease characterized by eosinophilia or neutrophilia-dominant airway inflammation were established by sensitization with OVA or OVA / LPS, respectively. Recombinant mouse IL-25 (rmIL-25) was administered intranasally on day-1, 6, 13, and 20 (Fig. 1A). H&E staining and inflammatory scoring of the lung sections showed obvious peri-bronchial infiltration of inflammatory cells in the mice sensitized with OVA or OVA / LPS compared with the control mice. IL-25 treatment significantly ameliorated the peri-bronchial infiltration of inflammatory cells and decreased the inflammatory scores of the mice sensitized with OVA / LPS (Fig. 1B, C). The total number of inflammatory cells in BALF from mice sensitized with OVA / LPS was markedly increased compared to mice sensitized with OVA and control mice, and the inflammatory cells were predominantly consisted of neutrophils (neutrophil 70.16%, eosinophil 0.47%, macrophage 24.30%, lymphocyte 4.52%). The number of eosinophils was significantly increased in BALF from mice sensitized with OVA (eosinophilia-dominant mice) compared with the mice treated with OVA / LPS (neutrophilia-dominant mice) and control mice. Intriguingly, intranasal administration of IL-25 significantly decreased the number of neutrophils in BALF from neutrophilia-dominant mice (Fig. 1D). Moreover, we found that the airway resistance to methacholine was increased in neutrophilia and eosinophilia-dominant mice compared to control mice. However, exogenous IL-25 markedly suppressed the airway resistance to methacholine in neutrophilia-dominant mice (Fig. 1E).

### IL-25 inhibits macrophage M1 polarization in the neutrophilia-dominant mouse model

**Fig. 2:**
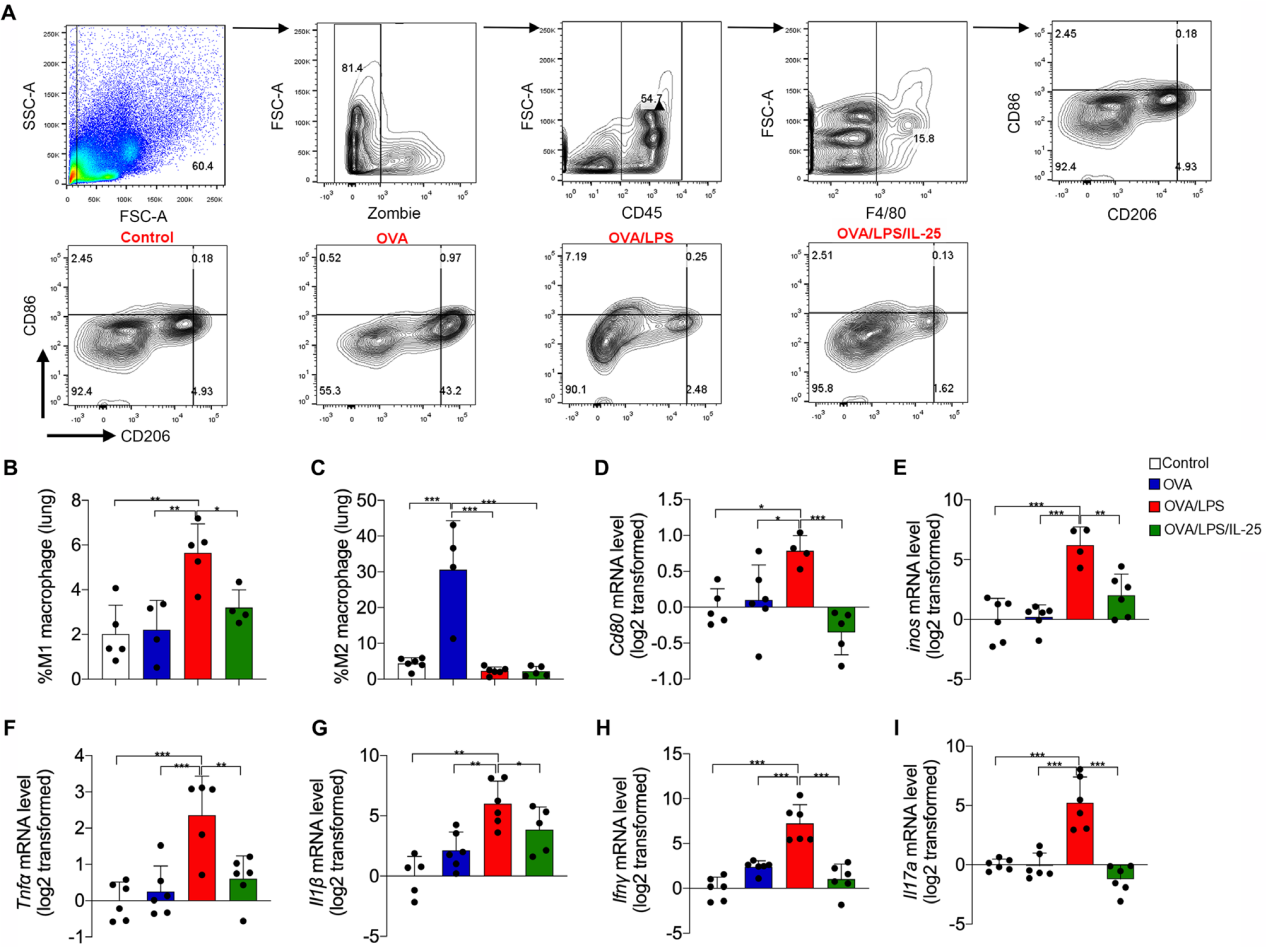
IL-25 inhibited macrophage M1 polarization in a mouse model of neutrophilia-dominant airway inflammation. (**A**) Flow cytometry of macrophages in mouse lung tissue. The percentages in the leftmost panel represents the proportion of monocytes, the second panel represents the proportion of living cells, the third panel represents the proportion of CD45^+^ living cells, the fourth panel represents the proportion of F4/80^+^ cells (macrophages). In the rightmost panel, CD45^+^F4/80^+^CD86^+^CD206^-^ represents M1 macrophage, and CD45^+^F4/80^+^CD86^-^CD206^+^ represents M2 macrophage; Representative dot plots showing the percentages of M1 and M2 macrophages in different groups. (**B**) The proportion of M1 macrophages in lung tissue of mice. (**C**) The proportion of M2 macrophages in lung tissue of mice. (**D**-**F**) Detection of Cd80, inos, Tnfα mRNA level in mouse lung tissue using RT-PCR. (**G**-**I**) Detection of Il1β, Ifnγ, and Il17a mRNA level in mouse lung tissue using RT-PCR. There were 4–6 mice in each group. One-way ANOVA was used for statistical analysis (* P < 0.05; ** P < 0.01; *** P < 0.001)

Macrophage M1 polarization was associated with airway neutrophilia in human asthma [11]. We next analyzed the macrophage polarization in mouse lung tissues and BAL cells by flow cytometry. Cd45^+^ F4/80^+^ cells were defined as macrophages, and Cd86 and Cd206 were used as markers for M1 and M2 macrophages, respectively (Fig. 2A). The percentage of Cd45^+^ F4/80^+^ CD86^+^ cells (M1-like macrophages) was significantly elevated in lung tissue of neutrophilia-dominant mice (5.56%) when compared with control mice (2.01%) or eosinophilia-dominant mice (2.20%). Of note, intranasal administration of rmIL-25 significantly decreased the percentage of M1-like macrophages in the lungs of neutrophilia-dominant mice (Fig. 2B). Similar results were observed in the BAL cells (Supplementary Fig. 1B). Cd45^+^ F4/80^+^ CD206^+^ cells (M2-like macrophages) account for 30.58% and 64.65%of total macrophages in lung tissue and in BAL cells from eosinophil-dominant mice, whereas accounts for only 2.29% and 0.03% of total macrophages in control and neutrophilia-dominant mice (Fig. 2C, Supplementary Fig. 1C). IL-25 treatment did not enhance the number of M2-like macrophages in neutrophilia-dominant mice. Consistent with the flow cytometry data, quantitative PCR revealed that the transcript levels of *Cd80* and *Inos*, markers for M1 macrophage, and the transcript levels of cytokines *Tnfα*, *Il1β*, *Ifnγ*, and *Il17* were increased in lungs from neutrophilia-dominant mice compared to control and eosinophilia-dominant mice, but were decreased after rmIL-25 treatment (Fig. 2D and I). These data suggest that M1-like macrophage may contribute to airway neutrophilia, and exogenous IL-25 may ameliorate airway neutrophilia by suppressing M1 polarization.

### IL-25 inhibits the expression of IL-12, IL-23 in the neutrophilia-dominant mouse model

**Fig. 3:**
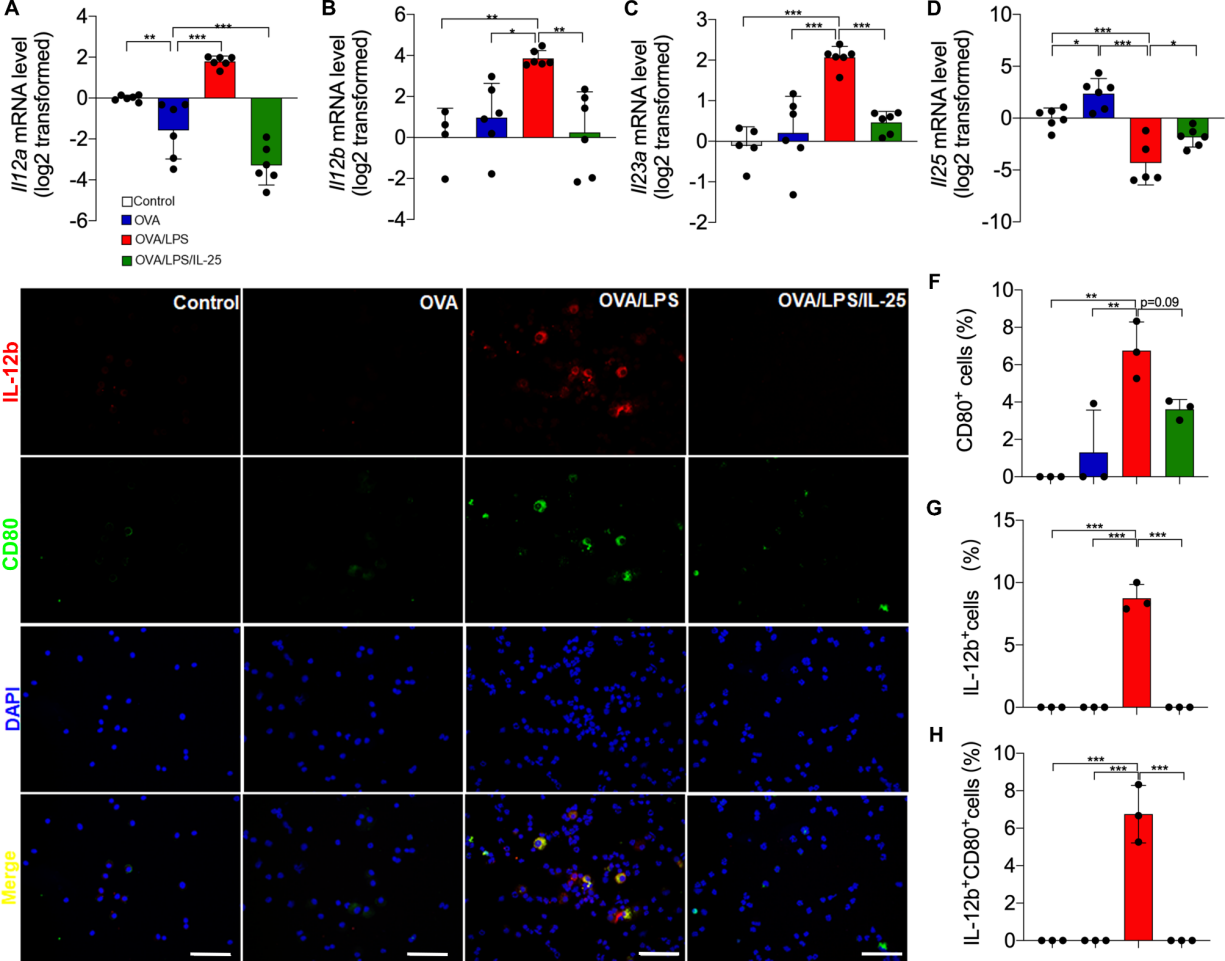
IL-25 inhibited the production of IL-12, IL-23 in amouse model of of neutrophilia-dominant airway inflammation. (**A**-**D**) Measurement of *Il12a*, *Il12b*, *Il23a* and *Il25* mRNA levels in mouse lung tissue using RT-PCR. (**E**) Representative images of IL-12B and CD80 immunofluorescence staining in mouse lung sections. Scale bar, 50 μm. (**F**) The proportion of CD80 staining-positive cells in mouse lung sections in different groups. (**G**) The proportion of IL-12B staining-positive cells in mouse lung sections in different groups. (**H**) The proportion of CD80 and IL-12B staining-positive cells in mouse lung sections in different groups. There were 4–6 mice in each group. One-way ANOVA was used for statistical analysis (* P < 0.05; ** P < 0.01; *** P < 0.001)

It has been reported that exogenous IL-25 suppresses intestinal inflammation by inhibiting IL-12 and IL-23 expression [20, 22]. IL-12 is a heterodimer of the subunits p35 (encoded by *IL-12 A*) and p40 (encoded by *IL-12B*), while IL-23 is composed of p19 (encoded by *IL-23 A*) and p40. We found that the transcript levels of *Il12a*, *Il12b*, and *Il23a* were increased in the lungs of neutrophilia-dominant mice compared to the eosinophilia-dominant mice or the control mice. Intranasal administration of rmIL-25 markedly suppressed the upregulation of *Il12a*, *Il12b*, and *Il23a* in the neutrophilia-dominant mice (Fig. 3A-C). The expression of *Il25* is increased in eosinophilia-dominant mice compared with control mice. Of note, *Il25* expression was decreased in neutrophilia-dominant mice when compared with control mice or eosinophilia-dominant mice (Fig. 3D). This indicates that the endogenous IL-25 expression is downregulated in the mouse model of neutrophilic airway inflammation. Moreover, the number of IL-12B staining-positive cells was markedly increased in BAL cells from neutrophilia-dominant mice when compared with control mice or eosinophil-dominant mice. Most of the IL-12B-positive cells (77.79%) were also positive for CD80, a marker for M1 macrophages. However, exogenous rmIL-25 treatment significantly decreased the number of IL-12B and CD80 staining-positive cells (Fig. 3E-H). Our data suggest that exogenous IL-25 may suppress IL-12 or IL-23 expression in macrophages.

### IL-25 inhibits macrophage M1 polarization and the expression of IL-12 and IL-23 in vitro

**Fig. 4:**
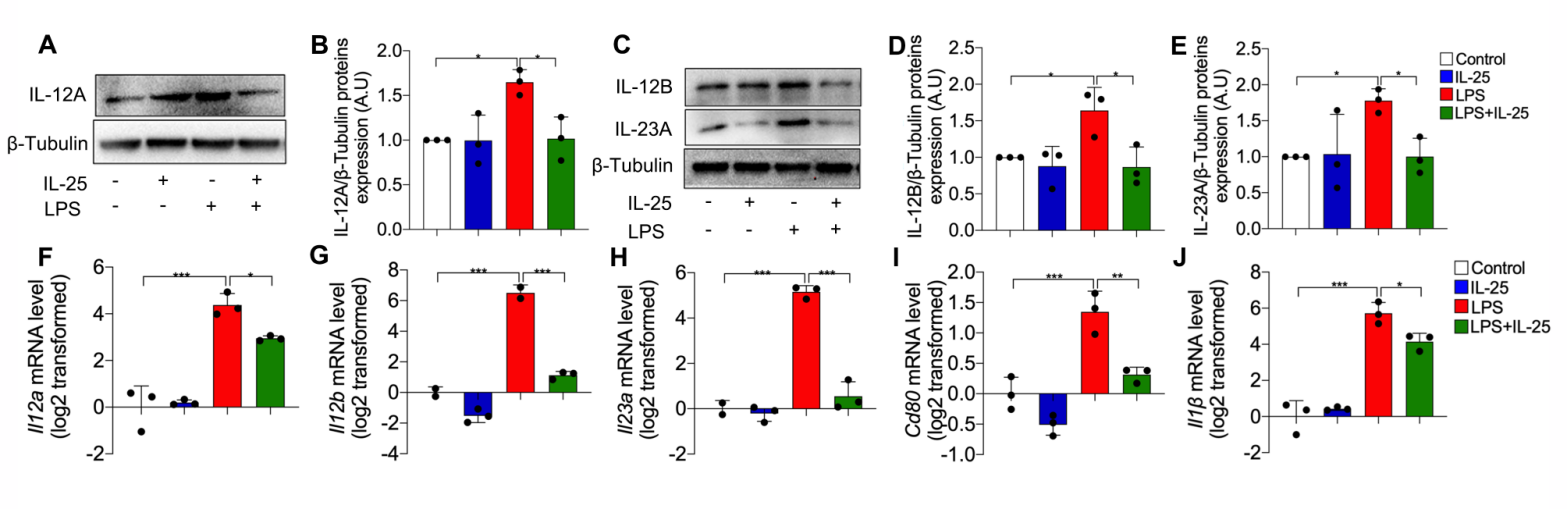
Exogenous IL-25 inhibited LPS-induced M1 polarization and the expression of IL-12 and IL-23 in mouse pulmonary cells. (**A**) Representative Western blots showing IL-12 A and β-Tubulin protein in primary culture of mouse pulmonary macrophages. (**B**) Quantitative analysis of IL-12 A in mouse pulmonary macrophages using ImageJ. Values are expressed in arbitrary units (a.u.). (**C**) Representative Western blots showing IL-12B, IL-23 A, and β-Tubulin protein in mouse pulmonary macrophages. (**D**-**E**) Quantitative analysis IL-12B, and IL-23A in mouse pulmonary macrophages using ImageJ. (**F**-**J**) Detection of *Il12a, Il12b, Il23a, Cd80*, and *Il-1β* mRNA level in mouse pulmonary macrophages using RT-PCR. The experiment was repeated 3 times independently, and a similar trend was obtained. One-way ANOVA was used for statistical analysis (* P < 0.05; ** P < 0.01; *** P < 0.001)

We next examined the effect of exogenous IL-25 on the expression of IL-12 and IL-23 in LPS-stimulated primary culture of macrophages from mice lungs. Western blotting revealed that IL-12 A, IL-12B, and IL-23 A protein levels were increased in LPS-stimulated macrophages compared to control cells. However, IL-25 (at a concentration of 100 ng/mL) markedly suppressed LPS-induced IL-12 A, IL-12B, and IL-23 A expression (Fig. 4A and E). Similar results were observed in the transcript levels of *Il12a*, *Il12b*, and *Il23a* of the primary macrophages (Fig. 4F-H). Moreover, exogenous IL-25 suppressed the LPS-induced *Cd80* and *Il1β* upregulation in macrophages (Fig. 4I, J). These in vitro data suggest that IL-25 suppresses macrophage M1 polarization and downregulates IL-12 and IL-23 expression.

### IL-25 suppresses LPS-induced c-Rel translocation to nuclei via STAT3 signaling

**Fig. 5:**
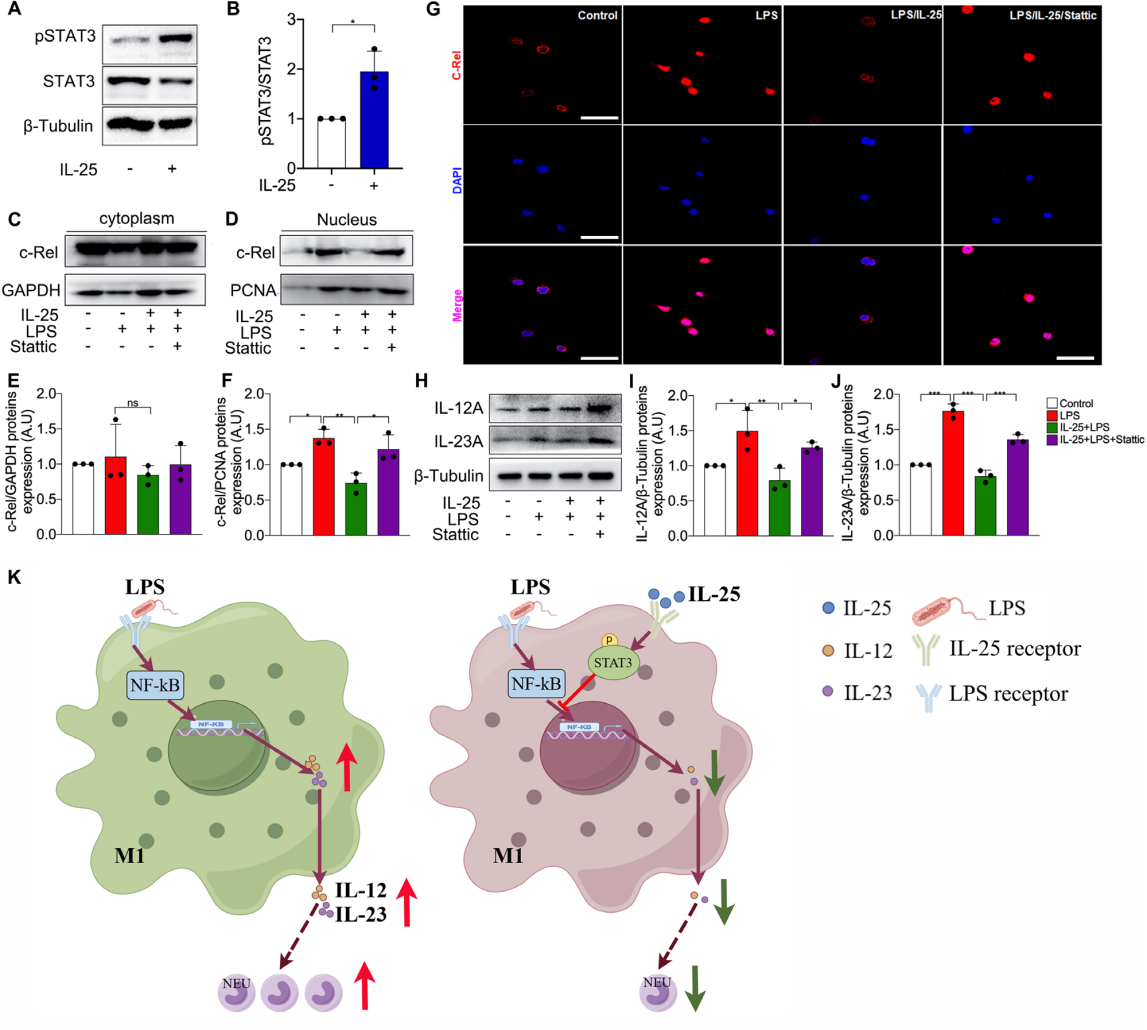
IL-25 inhibited LPS-induced translocation of c-Rel in STAT3-dependent manner in mouse pulmonary macrophages. (**A**) The level of STAT3 phosphorylation was detected using Western blot. (**B**) Quantitative analysis of STAT3 phosphorylation in primary culture of mouse pulmonary macrophages using ImageJ. Values are expressed in arbitrary units (a.u.). (**C**-**D**) Western blot was performed to measure the protein levels of c-Rel in the cytoplasm (**C**) and nucleus (**D**) of mouse pulmonary macrophages. (**E**-**F**) Quantitative analysis of c-Rel expresion in cytoplasm (**E**) and nucleus (**F**) of mouse pulmonary macrophages using ImageJ. (**G**) Representative images of c-Rel immunofluorescence staining in primary culture of macrophage from mouse lung. Scale bar, 50 μm. (**H**) The protein levels of IL-12 A and IL-23 A of mouse pulmonary macrophages was measured using Western blot. (**I**-**J**) Quantitative analysis of IL-12 A and IL-23 A in mouse pulmonary macrophages using ImageJ. Values are expressed in arbitrary units (a.u.). (**K**) The graphical abstract of this study, which was drawn by Figdraw. The experiment was repeated 3 times independently, and the similar trend was obtained. One way ANOVA was used for statistical analysis (* P < 0.05; ** P < 0.01; *** P < 0.001)

We further investigated the mechanism by which IL-25 inhibits the expression of IL-12 and IL-23. IL-25 can activate STAT3-mediated signaling [25–27]. Consistent with this, we found that IL-25 treatment increased the phosphorylation of STAT3 (Fig. 5A and B). The translocation of c-Rel from cytoplasm to nucleus is required for IL-12 expression in macrophages [18, 19]. We found that LPS stimulation increased the protein level of c-Rel in proteins from nuclei whereas IL-25 inhibited the LPS-induced c-Rel expression in nuclei (Fig. 5C-F). However, Stattic, a potent inhibitor of STAT3 phosphorylation, restored LPS-induced c-Rel upregulation in nuclei in the presence of exogenous IL-25 (Fig. 5C-F). Our data suggest that IL-25 inhibits LPS-induced c-Rel translocation from cytoplasm to nuclei through STAT3-mediated signaling. In line with the result of Western blotting, immunostaining of bronchoalveolar lavage (BAL) cells revealed that c-Rel was mainly expressed in the cytoplasm of control mice, but was expressed in the nuclei of the cells from LPS-treated neutrophil-dominant mice. IL-25 treatment suppressed LPS-induced c-Rel expression in nuclei whereas this effect is blocked by Stattic. Meanwhile, the inhibitory effect of IL-25 on the protein levels of IL-12 and IL-23 was also significantly weakened by Stattic (Fig. 5H-J).

### The expression of IL-25 was decreased whereas IL-12, IL-23 and M1 markers are increased in patients with severe asthma or non-eosinophilic asthma

**Fig. 6:**
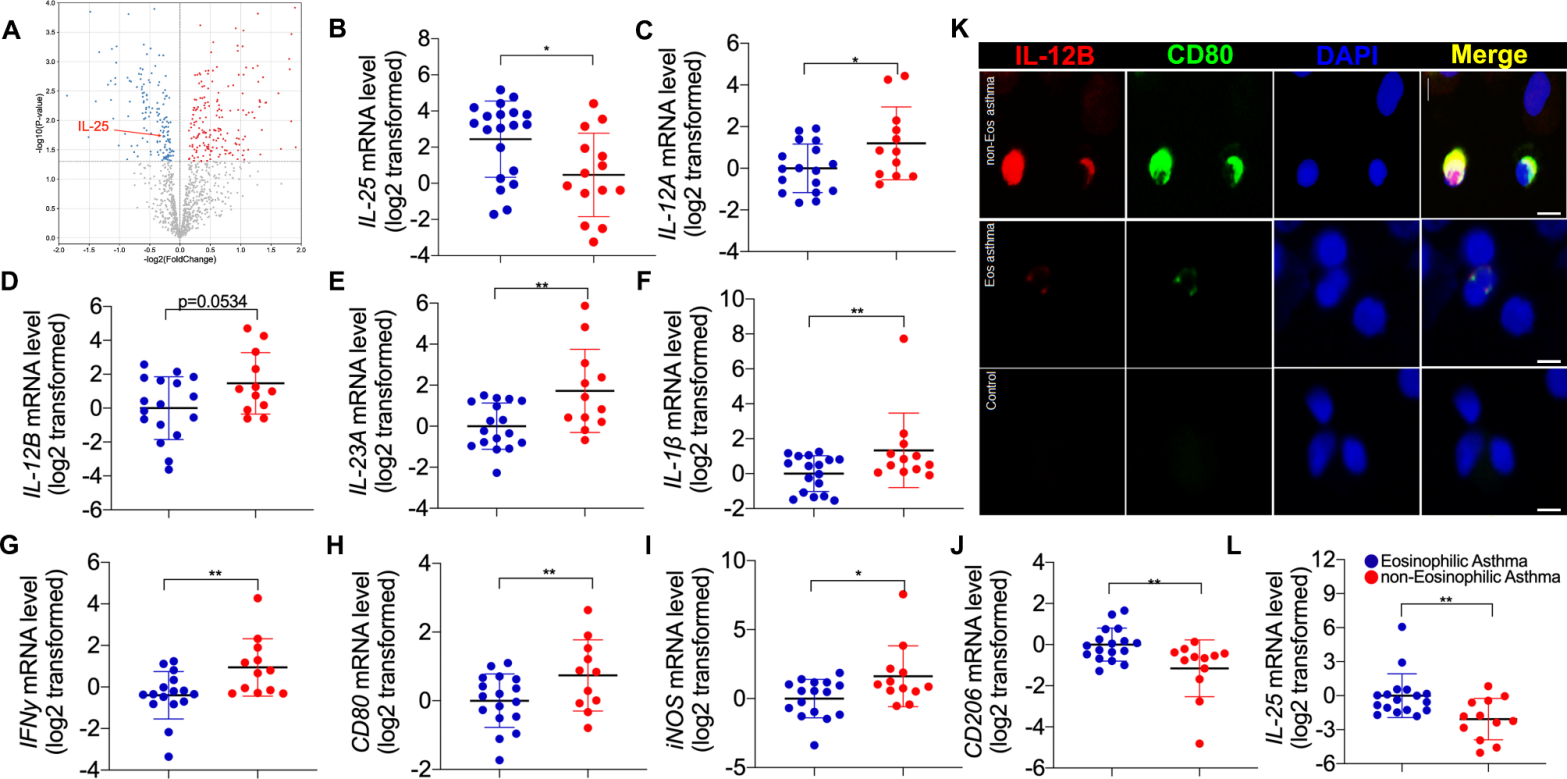
The expression of IL-25 was decreased whereas IL-12, IL-23, and M1 macrophage markers were increased in severe of non-eosinophilic asthmatics. (**A**) IL-25 protein levels were decreased in sputum from a cohort of non-smoker severe asthma in the U-BIOPRED study. Based on the analysis of the data provided by Takahashi et al. [28], 158 downregulated and 187 upregulated proteins were identified in the supernatant of induced sputum from non-smoker severe asthma patients (n = 37) compared to controls (n = 18) by proteomic assay and were shown in the volcano plot. The protein level of IL-25 (indicated by the red arrow) was decreased in the supernatant of induced sputum from non-smoker severe asthma patients compared to controls (log2 of fold change = -0.274, P = 0.019). (**B**) Detection of *IL-25* mRNA level in airway brushings of eosinophilic asthma (n = 20) and non-eosinophilic asthma (n = 14) by RT-PCR. (**C**-**E**) Detection of *IL-12 A, IL-12B* and *IL-23 A* mRNA level in induced sputum of eosinophilic asthma (n = 17) and non-eosinophilic asthma (n = 12) by RT-PCR. (**F**-**J**) Detection of I*L-1β, IFN-γ, CD80, iNOS*, and *CD206* mRNA level in induced sputum of eosinophilic asthma (n = 17) and non-eosinophilic asthma (n = 12) by RT-PCR. (**K**) Representative images of IL-12B and CD80 immunofluorescence staining in BALF cells of control, eosinophilic asthma, and non-eosinophilic asthma. Scale bar, 5 μm. (**L**) Detection of *IL-25* mRNA level in induced sputum of eosinophilic asthma (n = 17) and non-eosinophilic asthma (n = 12) by RT-PCR. One way ANOVA was used for statistical analysis. (* P < 0.05; ** P < 0.01; *** P < 0.001)

Based on our analysis of the data from a cohort with severe asthma in the U-BIOPRED study [28], the protein level of IL-25 was significantly decreased in sputum from non-smoker severe asthma (n = 37) compared to controls (n = 18) (Fig. 6A). This suggests that lack of endogenous IL-25 may contribute to pathogenesis of severe asthma. We further examined the expression of IL-12, IL-23 and IL-25 in bronchial epithelial brushings or induced sputum cells from our cohort of subjects with eosinophilic asthma (sputum eosinophils ≥ 3%), non-eosinophilic asthma (sputum eosinophil < 3%) and controls. The characteristics of the subjects are summarized (Table 1). There was no significant difference in age, sex ratio, body mass index, and FEV_1_% predicted and methacholine PD_20_ between the subjects with eosinophilic and non-eosinophilic asthma. Similar to the findings in sputum from severe asthma, the transcripts of *IL-25* in bronchial epithelial brushings were significantly lower in subjects with non-eosinophilic asthma (n = 14) than those with eosinophilic asthma (n = 20) (Fig. 6B). The transcripts of *IL12A*, and *IL23A* were significantly higher in sputum cells from subjects with non-eosinophilic asthma (n = 17) than those with eosinophilic asthma (n = 12) (Fig. 6C-E). We further analyze the expression of M1 markers including *CD80*, *iNOS*, and the cytokines derived from M1 macrophages including *IL-1β*, *IFNγ*. The transcript levels of these genes were significantly higher in sputum cells from subjects with non-eosinophilic asthma compared to eosinophilic asthma (Fig. 6F-I), suggesting M1 macrophages may be predominant in the sputum cells from non-eosinophilic asthma. In support of this, immunostaining revealed that the existence of IL-12B^+^ CD80^+^ cells in BAL cells from subjects with non-eosinophilic asthma (Fig. 6K). In addition, IL-25 transcripts were lower in sputum cells from subjects with non-eosinophilic asthma than those with eosinophilic asthma (Fig. 6L). Our data suggest that the decreased expression of endogenous IL-25 in severe asthma or non-eosinophilic asthma is associated with increased expression of IL-12, IL-23, and M1 markers.

**Table 1:** Subject characteristics for induced sputum

	Controls	Eosinophilic asthma	non-Eosinophilic asthma	P value (Control vs. asthma)	P value (Eos vs. non-Eos)
Number	10	17	12		
Age, y	37.78 ± 6.56	38.38 ± 6.62	44.25 ± 9.08	0.0992	0.0676
Sex, M: F, %F	6:4, 60.00	6:11, 64.71	3:9, 75	0.243	0.694
Body mass index	21.89 ± 2.23	23.33 ± 1.55	23.53 ± 1.74	0.1908	0.7911
FEV_1_, %predicted	99.04 ± 10.63	85.20 ± 13.08	85.77 ± 12.13	0.033	0.9144
Methacholine PD_20_, mg	2.505 ± 0	0.275 ± 0.409	0.392 ± 0.407	< 0.0001	0.5316
Sputum eosinophil, %	0.45 ± 0.66	20.47 ± 19.97	2.61 ± 2.53	0.0007	0.0062
Sputum neutrophil, %	13.62 ± 18.55	29.52 ± 19.33	35.35 ± 24.85	0.0705	0.4993
Sputum macrophage, %	83.35 ± 19.68	43.55 ± 25.58	54.23 ± 23.47	0.001	0.2788

## Discussion

In the present study, we demonstrated that IL-25 expression was decreased in sputum from subjects with severe asthma, sputum cells from subjects with non-eosinophilic asthma, and in a mouse model of neutrophilia-dominant airway inflammation. In contrast, the markers of M1 macrophages and the expression of IL-12 and IL-23 were increased in sputum cells from non-eosinophilic asthma, and the percentage of M1 macrophages was enhanced in the neutrophilia-dominant mouse model. Importantly, exogenous IL-25 markedly decreased the number of M1 macrophages and the expression of IL-12 and IL-23, and ameliorated airway neutrophilic inflammation in the neutrophilia-dominant mice. Our findings suggest that IL-25 expression is downregulated in non-eosinophilic asthma and exogenous IL-25 ameliorates airway neutrophilia, at least in part, via suppressing macrophage M1 polarization and the expression of IL-12 and IL-23.

We previously reported that epithelial IL-25 expression is associated with type 2 status and responsiveness to inhaled corticosteroids in asthma [29]. In this study, we demonstrated that IL-25 protein levels were decreased in sputum from a cohort of nonsmoker severe asthma in the U-BIOPRED study [28]. Moreover, IL-25 expression was decreased in sputum cells from non-eosinophilic asthma compared to eosinophilic asthma. These observations suggest that the downregulation of IL-25 may contribute to the pathogenesis of severe asthma or non-eosinophilic asthma. Interestingly, IL-25 expression was also decreased in the lungs from the mouse model of neutrophilia-dominant airway inflammation.

Non-eosinophilic asthma is associated with macrophage M1 activation. Kim and colleagues reported that the percentages of neutrophils were positively correlated with the percentage of M1 macrophages in induced sputum from subjects with non-eosinopihlic asthma [11]. Consistent with this, we found that the expression of M1 markers including *CD80*, *iNOS*, and the cytokines derived from M1 macrophages including *IL-1β*, *IFNγ* was higher in the sputum cells from non-eosinophilic asthma than in eosinophilic asthma. Moreover, the percentage of M1 macrophages was higher in the lung of a mouse model of neutrophilia-dominant allergic airway inflammation. Moreover, the expression of IL-12B was increased in the BAL cells of this model.

M1 macrophage can express proinflammatory cytokines IL-12 and IL-23, and the upregulation of both cytokines were associated with human subjects with severe asthma and mouse models of severe asthma [15]. Interestingly, the expression of IL-12 and IL-23 were increased in the intestine of *Il25*^−/−^ mice, suggesting that endogenous IL-25 is required to limit the expression of IL-12 and IL-23 [20]. It was reported that IL-25 suppressed the expression of IL-6, IL-23, and IL-1β in LPS-induced M1 macrophages in vitro, and IL-25 deficiency led to M1 polarization in murine models of trachea transplantation [30]. Here, we showed that IL-25 inhibits macrophage M1 activation and the expression of IL-12 and IL-23, and ameliorates airway neutrophilia in the mouse model of neutrophilic-dominant allergic airway inflammation. In the mechanistic experiment using primary culture of macrophages from mice lungs, we demonstrated that IL-25 inhibits macrophage M1 activation via suppressing c-Rel translocation to nuclei in a STAT3-dependent manner.

Our study has several limitations. Because IL-25 can act on other immune cells including ILC2, Th2, and eosinophil [31–33], we cannot exclude that IL-25 may ameliorate airway neutrophilia via macrophage-independent mechanism. Other studies and ours demonstrated the association between macrophage M1 activation and airway neutrophilia in human asthma and animal models. However, future studies are required to investigate the contribution of macrophage M1 activation to airway neutrophilia in severe or neutrophilic asthma.

## Conclusion

Our study suggests that IL-25 expression is downregulated in severe or non-eosinophilic asthma. IL-25 acts as a negative regulator of the neutrophilic airway inflammation, at least in part, via suppressing macrophage M1 activation and the expression of IL-12 and IL-23. Exogenous IL-25 may represent a potential therapy for neutrophilic asthma.

### Electronic supplementary material

Below is the link to the electronic supplementary material.

Additional file 1: Figure S1

## Data Availability

All data generated or analyzed during this study are included in this published article and its supplementary information files.

## Abbreviations

PD_20_: Dosage of methacholine provoking a 20% fall
FEV_1_: Forced expiratory volume in the first second
BALF: Bronchoalveolar lavage fluid
H&E: Hematoxylin and eosin
PAS: Periodic acid-Schiff
PVDF: Polyvinylidene difluoride
ELISA: Enzyme linked immunosorbent assay
SD: Standard deviation
