# Supplementary material for: Exogenous IL-25 ameliorates airway neutrophilia via suppressing macrophage M1 polarization and the expression of IL-12 and IL-23 in asthma
Source: Respir Res. 2023 Oct 28;24:260. doi: 10.1186/s12931-023-02557-5 (PMC10613395; doi:10.1186/s12931-023-02557-5)
Supplement: Supplementary file 1 — Additional file 1: Figure S1 [file 12931_2023_2557_MOESM1_ESM.docx]

Supplementary 1.


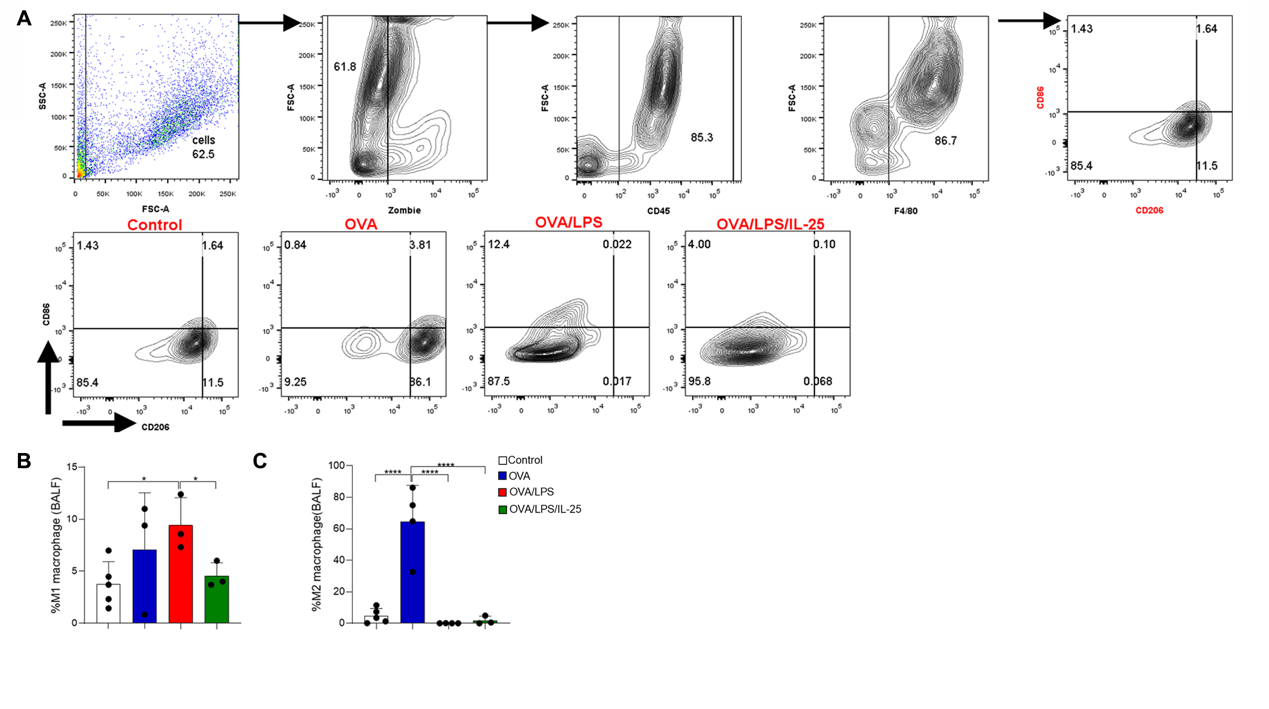


**Il-25 inhibited macrophage M1 polarization in the mouse model of neutrophilic asthma.**

A, Flow cytometry of macrophages in BALF of mice. The percentages in the leftmost panel represents the proportion of monocytes, the second panel represents the proportion of living cells, the third panel represents the proportion of CD45^+^ living cells, and the fourth panel represents the proportion of F4/80+ cells (macrophages). In the rightmost panel, CD45^+^F4/80^+^CD86^+^CD206^-^ cells represent M1 macrophage, and CD45^+^F4/80^+^CD86^-^CD206^+^ cells represent M2 macrophage; Representative dot plots showing the percentages of M1 and M2 macrophages in different groups. B, The proportion of M1 macrophages in BALF of mice. C, The proportion of M2 macrophages in BALF of mice.
